# Immunometabolic factors in adolescent chronic disease are associated with Th1 skewing of invariant Natural Killer T cells

**DOI:** 10.1038/s41598-021-99580-7

**Published:** 2021-10-11

**Authors:** Francesca A. Ververs, Suzanne E. Engelen, Roos Nuboer, Bas Vastert, Cornelis K. van der Ent, Belinda van’t Land, Johan Garssen, Claudia Monaco, Marianne Boes, Henk S. Schipper

**Affiliations:** 1 Center for Translational Immunology, University Medical Center Utrecht, Utrecht, The Netherlands; 2 Kennedy Institute of Rheumatology, University of Oxford, Oxford, UK; 3 Department of Pediatrics, Meander Medical Center Amersfoort, Amersfoort, The Netherlands; 4 Department of Pediatric Rheumatology and Immunology, Wilhelmina Children’s Hospital, University Medical Center Utrecht, Utrecht, The Netherlands; 5 Department of Pediatric Pulmonology, Wilhelmina Children’s Hospital, University Medical Center Utrecht, Utrecht, The Netherlands; 6 Center of Excellence Immunology, Danone Nutricia Research, Utrecht, The Netherlands; 7 Division Pharmacology, Utrecht Institute for Pharmaceutical Sciences, Beta Faculty, Utrecht University, Utrecht, The Netherlands; 8 Department of Pediatric Cardiology, Wilhelmina Children’s Hospital, University Medical Center Utrecht, Utrecht, The Netherlands

**Keywords:** Juvenile idiopathic arthritis, Dyslipidaemias, Chronic inflammation, Lymphocyte activation, Cystic fibrosis, Obesity

## Abstract

Invariant Natural Killer T (iNKT) cells respond to the ligation of lipid antigen-CD1d complexes via their T-cell receptor and are implicated in various immunometabolic diseases. We considered that immunometabolic factors might affect iNKT cell function. To this end, we investigated iNKT cell phenotype and function in a cohort of adolescents with chronic disease and immunometabolic abnormalities. We analyzed peripheral blood iNKT cells of adolescents with cystic fibrosis (CF, n = 24), corrected coarctation of the aorta (CoA, n = 25), juvenile idiopathic arthritis (JIA, n = 20), obesity (OB, n = 20), and corrected atrial septal defect (ASD, n = 25) as controls. To study transcriptional differences, we performed RNA sequencing on a subset of obese patients and controls. Finally, we performed standardized co-culture experiments using patient plasma, to investigate the effect of plasma factors on iNKT cell function. We found comparable iNKT cell numbers across patient groups, except for reduced iNKT cell numbers in JIA patients. Upon *ex-vivo* activation, we observed enhanced IFN-γ/IL-4 cytokine ratios in iNKT cells of obese adolescents versus controls. The Th1-skewed iNKT cell cytokine profile of obese adolescents was not explained by a distinct transcriptional profile of the iNKT cells. Co-culture experiments with patient plasma revealed that across all patient groups, obesity-associated plasma factors including LDL-cholesterol, leptin, and fatty-acid binding protein 4 (FABP4) coincided with higher IFN-γ production, whereas high HDL-cholesterol and insulin sensitivity (QUICKI) coincided with higher IL-4 production. LDL and HDL supplementation in co-culture studies confirmed the effects of lipoproteins on iNKT cell cytokine production. These results suggest that circulating immunometabolic factors such as lipoproteins may be involved in Th1 skewing of the iNKT cell cytokine response in immunometabolic disease.

## Introduction

Invariant Natural Killer T (iNKT) cells are a unique innate-like T cell subset that respond to lipid antigens presented on the MHCI-like molecule CD1d, and operate on the border of metabolic derangement and inflammation^1^. As such, they have been implicated in immunometabolic diseases such as obesity, type II diabetes, and cardiovascular disease^1^. iNKT cells bear diagnostic and therapeutic potential because they can produce copious amounts of anti-inflammatory and pro-inflammatory cytokines, either steering towards a regulatory or inflammatory adaptive immune response^2^. Understanding the mechanisms driving a regulatory versus inflammatory iNKT cell response is crucial for potential diagnostic or therapeutic use of iNKT cells, but is complicated by the fact that the involved iNKT cell antigens remain elusive^3^. The CD1d-binding affinity of lipid antigens seems to affect iNKT cell function^3^. Longer acyl chains with greater binding affinity allow for a longer interaction between the antigen presenting cell (APC) and iNKT cell, which enables upregulation of fate-determining co-stimulatory molecules^1^. Next to lipid antigens, the type and activation state of the APC and tissue microenvironment affect the iNKT cell cytokine response^4–6^. For example in adipose tissue, iNKT cells respond to lipid antigens presented by adipocytes as non-professional APCs. In lean adipose tissue iNKT cells produce regulatory cytokines such as IL-4 and IL-13. During obesity, iNKT cells respond to lipids presented by hypertrophied adipocytes with a more pro-inflammatory cytokine response, contributing to adipose tissue inflammation and ensuing insulin resistance^6–9^. In addition to antigenic and environmental factors, there are intrinsic differences between iNKT cell subsets that contribute to iNKT cell functional diversity^10,11^. iNKT cell functional subsets are defined analogous to T helper subsets. Th1 iNKT cells in humans are associated with a CD8 positive or double negative (DN) phenotype and IFN-γ production, while Th2 iNKT cells are associated with CD4 expression and IL-4 and IL-13 production^6^.

In this study we investigated whether immunometabolic factors in chronic disease may also affect iNKT cell phenotype and function in a diverse cohort of adolescents with inflammatory, metabolic, and hemodynamic abnormalities. We analyzed iNKT cells from patients previously participating in the *“Cardiovascular Disease in Adolescents with Chronic Disease”* (CDACD) study. The study included adolescents with cystic fibrosis (CF) and obesity (OB), associated with characteristic metabolic and inflammatory abnormalities, corrected coarctation of the aorta (CoA) frequently coinciding with hypertension, juvenile idiopathic arthritis (JIA) associated with chronic inflammation, and a healthy control group with a history of atrial septal defect (ASD). We analyzed circulating iNKT cells using flow cytometry and measured the *ex-vivo* cytokine response upon α-galactosylceramide (α-GalCer) stimulation. Next, we performed low-input RNA sequencing in a subset of obese adolescents and controls, to uncover transcriptional differences. Finally, we performed standardized co-culture experiments using THP-1 monocytes loaded with patient plasma as antigen presenting cells, in co-culture with healthy donor-derived short-term iNKT cell lines, to investigate the effect of plasma factors on iNKT cell function. These experiments were followed by LDL and HDL supplementation studies. By analyzing iNKT cells in such a diverse cohort of adolescents with a range of immunometabolic abnormalities, we aimed to identify which inflammatory and metabolic factors are associated with skewing of the iNKT cell phenotype.

## Results

### Immunometabolic profiles of adolescents with chronic disease

Adolescents from various chronic disease groups and representing a range of inflammatory, metabolic, and hemodynamic abnormalities were recruited in order to study the relation between immunometabolic factors and iNKT cell phenotype and function. Compared to healthy ASD controls, CF patients showed low cholesterol and insulin levels, in line with a CF pancreatic insufficiency phenotype. Furthermore, CF is associated with visceral adipose tissue accumulation, which was reflected by a high waist-to-hip ratio (WHR) (Table 1)^12^. CoA patients showed elevation of systolic blood pressure (SBP) characteristic for this patient population, and a higher WHR, which may be explained by the male predominance in this group. Adolescents with JIA had a history of polyarticular or extended oligoarticular juvenile idiopathic arthritis, but most JIA patients were in remission during the study and did not show active signs of systemic inflammation. Finally, obese adolescents showed characteristic features including a higher body mass index (BMI-SD) and WHR, lower insulin sensitivity (QUICKI), dyslipidemia with high fasting triglycerides and low HDL-cholesterol, and elevated high-sensitivity CRP levels reflecting low-grade systemic inflammation (Table 1). In summary, adolescents with chronic disease showed characteristic phenotypes, with distinct metabolic abnormalities in adolescents with CF, high systolic blood pressure in CoA patients, and combined metabolic and inflammatory abnormalities in the obese adolescents.

**Table 1:** Immunometabolic profiles of adolescents with chronic disease.

	ASD	CF	CoA	JIA	OB
N (m/f)	25 (3/22)	24 (13/11)**	25 (17/8)***	20 (6/14)	20 (8/12)*
Age (years)	14.32 (12.66–17.02)	15.92 (14.18–17.29)	14.55 (12.73–16.46)	16.10 (13.82–16.95)	14.61 (12.99–16.72)
BMI (SD)	− 0.15 ± 0.99	− 0.36 ± 0.93	0.19 ± 1.26	0.07 ± 1.06	3.23 ± 0.33***
Waist-to-hip ratio	0.76 (0.73–0.79)	0.84 (0.78–0.89)**	0.81 (0.77–0.85)*	0.79 (0.73–0.83)	0.91 (0.83–0.96)***
***Glucose***					
Fasting glucose (mmol/L)	5.00 (4.90- 5.30)	5.25 (4.83- 5.73)	5.20 (4.95- 5.30)	5.10 (4.90- 5.58)	5.20 (4.93- 5.48)
Fasting insulin (mmol/L)	9.50 (8.05- 14.00)	7.40 (5.60- 9.80)*	9.30 (6.40- 10.00)	9.85 (8.43- 14.00)	21.00 (13.75- 31.25)***
QUICKI	0.34 ± 0.02	0.35 ± 0.03	0.35 ± 0.02	0.33 ± 0.02	0.30 ± 0.02***
***Lipids***					
LDL-cholesterol (mmol/L)	2.10 (1.70–3.10)	1.50 (1.13–2.08)	2.40 (1.90–2.70)	2.25 (1.93–2.50)	2.49 (2.20–3.10)
HDL-cholesterol (mmol/L)	1.37 ± 0.22	1.16 ± 0.22**	1.27 ± 0.23	1.28 ± 0.25	1.22 ± 0.21*
Triglycerides (mmol/L)	0.80 (0.50–0.90)	0.80 (0.60–0.90)	0.80 (0.65–1.05)	0.80 (0.60–1.00)	1.10 (1.00–1.40)***
***Inflammation***					
Hs- CRP (mg/L)	0.86 (0.38–5.97)	3.94 (0.92–13.73)	1.14 (0.42–4.16)	1.16 (0.45–4.67)	9.24 (5.58–26.49)**
Lymphocytes (× 10^9^/L)	2.06 ± 0.67	2.09 ± 0.68	1.87 ± 0.54	1.87 ± 0.48	2.42 ± 0.69
Monocytes (× 10^^9^/L)	0.50 (0.44–0.56)	0.52 (0.35–0.66)	0.46 (0.42–0.56)	0.47 (0.38–50)	0.48 (0.40–0.56)
***Adipokines***					
FABP4 (ng/mL)	9.61 (8.04–15.73)	9.69 (5.79–12.65)	10.57 (5.32–14.13)	11.77 (8.90–16.03)	17.64 (11.79–24.96)*
Adiponectin (μg/mL)	131.28 (113.23–142.54)	122.99 (100.30–151.02)	105.37 (90.69–133.13)*	120.12 (93.95–154.39)	104.60 (78.74–128.95)*
Leptin (ng/mL)	1.08 (0.66–2.14)	0.50 (0.18–1.06)*	0.58 (0.13–1.18)*	1.10 (0.20–2.94)	8.38 (4.48–10.92)***
Chemerin (ng/mL)	61.44 (50.81–68.24)	58.02 (49.75–65.15	62.09 (52.23–70.90)	62.54 (51.52–67.81)	61.99 (55.73–88.39)
MCP-1/CCL-2 (pg/mL)	37.62 (19.82–60.89)	39.63 (27.43–71.31)	50.02 (27.30–58.98)	37.19 (25.14–62.58)	31.17 (21.66–42.95)
Cathepsin S (ng/mL)	49.60 ± 1.82	53.79 ± 2.16	49.67 ± 2.26	52.71 ± 2.02	46.64 ± 1.99

ASD: atrial septal defect, CF: cystic fibrosis, CoA: coarctation of the aorta, JIA: juvenile idiopathic arthritis, OB: obesity, BMI (SD): body mass index standard deviation from the age- and sex matched population mean, QUICKI: quantitative insulin sensitivity check index, LDL: low-density-lipoprotein cholesterol, HDL: high-density-lipoprotein cholesterol, hs-CRP: high sensitivity C-reactive protein, FABP4: fatty acid binding protein 4, MCP-1: monocyte chemoattractant protein 1, CCL-2: C–C motif chemokine ligand 2. All chronic disease groups were compared to healthy ASD controls. * p < 0.05, ** p < 0.01, *** p < 0.001.

### Obese adolescents show Th1 skewing of activated circulating iNKT cells

**Figure 1:**
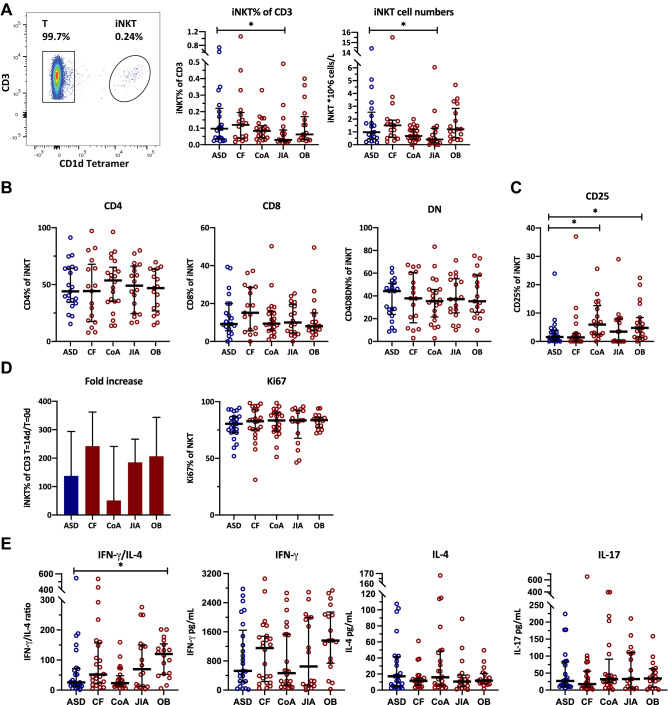
iNKT cell numbers and phenotype. (**A**) Circulating iNKT cell numbers presented as percentage of CD3 and as absolute numbers, measured using flow-cytometry of PBMC from patients with CF (n = 17), CoA (n = 22), JIA (n = 18), and obesity (n = 17), and compared with ASD controls (n = 17) using Kruskal–Wallis, followed by post-hoc Mann–Whitney U tests, *p < 0.05. Differences were not significant after multiple testing correction. (**B**) CD4, CD8 and double negative (DN) iNKT subset division. (**C**) iNKT cell CD25 expression at T = 0. The observed differences were not significant after multiple testing correction. (**D**) iNKT cell proliferation and Ki-67 expression after 14 days culture following α-GalCer stimulation. (**E**) iNKT cell cytokine production measured in supernatant using multiplex immunoassay, after 11 days culture following α-GalCer stimulation of PBMC from patients with CF (n = 24), CoA (n = 24), JIA (n = 18), and obesity (n = 18), compared with ASD controls (n = 25) using Kruskal–Wallis, followed by post-hoc Mann–Whitney U tests, * p < 0.05. Differences remained significant after multiple testing correction.

Baseline assessment of iNKT cell numbers and phenotype using flow-cytometry revealed slightly lower iNKT cell numbers in the circulation of adolescents with JIA compared to ASD controls, but not in adolescents with CF, CoA, or obesity (Fig. 1A). There were no differences in the CD4/CD8/double negative (DN) iNKT cell subset divisions (Fig. 1B). Obese and CoA patients did show a higher baseline expression of the iNKT cell activation marker CD25 (Fig. 1C). Upon α-GalCer stimulation, iNKT cells of all groups showed similar proliferation and Ki-67 expression (Fig. 1D and supplementary figure S1). There were no differences in IL-17, IL-4 or IFN-γ cytokine production, except for a higher IFN-γ/IL-4 cytokine ratio in obese adolescents compared to ASD controls, indicating a Th1-skewed pro-inflammatory phenotype (Fig. 1E). Considering that differences in iNKT cell function can be induced by APCs^4^, we also phenotyped the main circulating APC subsets. Obese adolescents showed a lower CD1d expression on nonclassical monocytes than controls, but similar CD1d expression of the other monocyte subsets, B cells and dendritic cells (supplementary figure S2A). Furthermore, B cells from obese patients showed a higher expression of adhesion molecule CD62L (supplementary figure S2B). Taken together, obese patients showed a distinct iNKT cell phenotype compared to the other groups, with higher baseline expression of CD25 and skewing of IFN-γ over IL-4 production upon α-GalCer stimulation, which could not be explained by differences in the circulating APC subsets. After correction for multiple testing, only the higher IFN-γ/IL-4 cytokine ratio in obese adolescents compared to ASD controls remained significant (Fig. 1E).

### iNKT cells from obese adolescents do not show a distinct transcriptional profile

**Figure 2:**
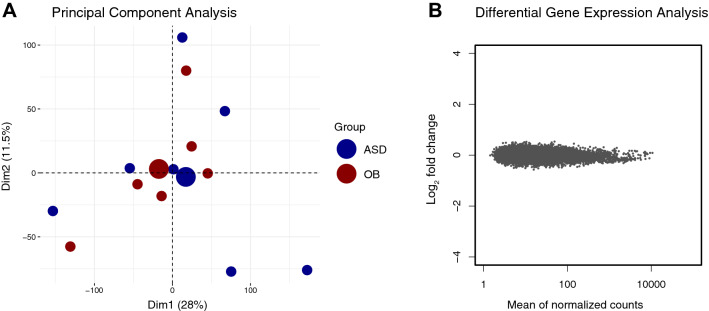
Transcriptional profile of iNKT cells from obese adolescents versus controls. (**A**) Principal component (PC) analysis of gene expression based on log2-transformed reads per million (RPM) following RNA sequencing, obese adolescents (n = 7) are red and age-and sex matched ASD controls are blue (n = 7). (**B**) MA plot showing no differential iNKT cell gene expression between obese adolescents and ASD controls (False Discovery Rate-corrected *p* < 0.01).

In order to establish whether intrinsic differences in iNKT cell gene expression could account for IFN-γ/IL-4 cytokine skewing in obese adolescents, iNKT cells from a subset of obese adolescents and age-and sex matched healthy ASD controls were isolated for low-input RNA sequencing (CEL-seq). RNA sequencing revealed that there were no differentially expressed genes between iNKT cells from obese adolescents versus lean controls (Fig. 2A and 2B). Allowing a more flexible cut-off using a non-corrected nominal p-value of 0.01, revealed the top 48 differentially expressed genes (supplementary figure S3). However, among the top differentially expressed genes, no biologically meaningful pattern or pathways could be identified, even when widening the cut off to a nominal p-value of 0.05. In addition, no relative gene set enrichment was observed in either iNKT cells from obese adolescents or ASD controls using gene set enrichment analysis (GSEA). In summary, the distinct iNKT cell phenotype and function of obese adolescents could not be explained by intrinsic iNKT cell differences.

### Obesity-associated plasma factors are associated with Th1 skewing in immunometabolic disease

**Figure 3:**
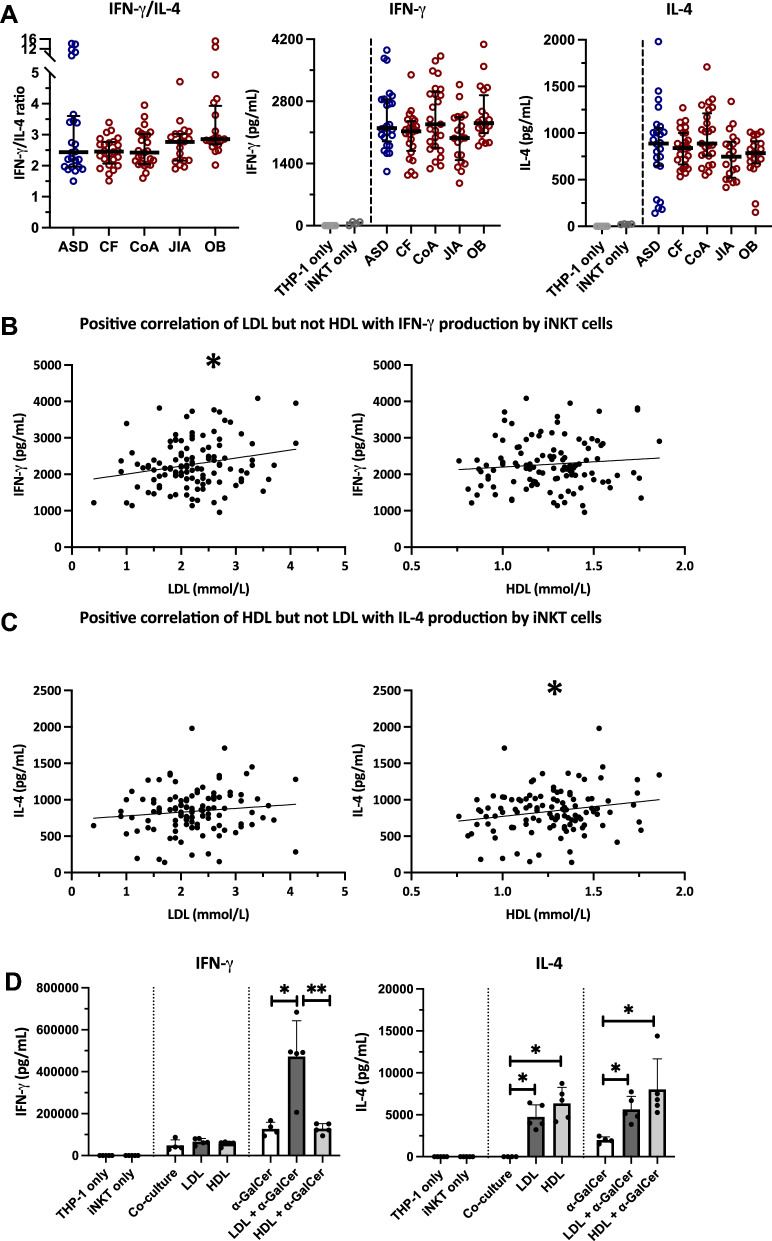
Plasma factors affect the iNKT cell phenotype. **(A**) Plasma-induced iNKT cell cytokine production in co-culture measured using ELISA (n = 114), next to THP-1 only (n = 6) and iNKT cells only (n = 4) controls. Plasma was obtained from patients and added to a standardized co-culture set-up using a THP-1 cell line and short-term iNKT cell line. Disease groups were compared using Kruskal–Wallis, followed by post-hoc Mann–Whitney U tests against ASD controls, * p < 0.05. (**B**) IFN-γ production corresponded with LDL-cholesterol levels in plasma (n = 114, Pearson’s R = 0.222, p = 0.017) but not HDL-cholesterol (n = 114, Pearson’s R = 0.100, p = 0.288). (**C**) IL-4 cytokine production corresponded with HDL-cholesterol levels in plasma (n = 114, Pearson’s R = 0.206, p = 0.029) but not LDL-cholesterol (n = 114, Pearson’s R = 0.115, p = 0.224). (**D**) Lipoprotein-induced iNKT cell cytokine production in co-culture. THP-1 macrophages were treated overnight with human LDL or HDL with or without α-GalCer. THP-1 cells were washed before human iNKT cells were added for 24 h (n = 5, for controls n = 4). Co-culture supernatants were harvested and analyzed for human IFN-γ and IL-4 using ELISA, and compared using Mann–Whitney U tests, * p < 0.05, ** p < 0.01. Differences remained significant after multiple testing correction.

Finally, we considered whether immunometabolic plasma factors were associated with the Th1 skewing of the iNKT cells in obese adolescents. We examined the role of patient plasma factors in a standardized co-culture system. Upon loading of THP-1 monocytes with patient-derived plasma, followed by a co-culture with healthy donor-derived short-term iNKT cell lines, we observed a similar trend of IFN-γ/IL-4 cytokine skewing using plasma of obese adolescents compared to controls (p = 0.08) (Fig. 3A). Multivariable linear regression analysis across the chronic disease groups identified the immunometabolic plasma factors involved (Table 2), and revealed that IFN-γ production by iNKT cells was primarily associated with sex, BMI (SD), LDL-cholesterol, FABP4, and leptin, whereas IL-4 production was associated with HDL-cholesterol and QUICKI (Table 2). The association between the iNKT cell IFN-γ response and higher plasma LDL-cholesterol and leptin levels, as well as the association between the iNKT cell IL-4 response and higher HDL-cholesterol and QUICKI, remained after exclusion of the obese adolescents from the analysis (supplementary table S1). Univariate correlation analyses confirmed that a higher LDL-cholesterol plasma content corresponded with higher IFN-γ production (Fig. 3B, supplementary figure S4). Likewise, higher HDL-cholesterol plasma levels coincided with higher IL-4 production (Fig. 3C). We hypothesized that lipoproteins could affect iNKT cell activation by the APC, and performed in vitro supplementation studies of LDL and HDL. THP-1 macrophages were pre-treated with human LDL or HDL, with and without α-GalCer, and subsequently co-cultured with short-term human iNKT cell lines. Pre-treatment of the THP-1 macrophages with LDL-cholesterol resulted in significantly higher α-GalCer-induced IFN-γ production by the iNKT cells, compared to α-GalCer alone or α-GalCer together with HDL-cholesterol (Fig. 3D). In contrast, the IL-4 production by iNKT cells was enhanced by LDL and even more by HDL supplementation, with and without α-GalCer loading of the THP-1 macrophages (Fig. 3D). These differences remained significant after multiple testing correction. In conclusion, obesity-associated immunometabolic factors including LDL-cholesterol, FABP4, and leptin were associated with a higher iNKT cell IFN-γ response across all disease groups, while HDL-cholesterol and insulin sensitivity (QUICKI) were associated with a higher IL-4 response. In vitro supplementation studies confirmed the cytokine skewing of iNKT cells by LDL and HDL.

**Table 2:** Multivariable linear regression identifies immunometabolic predictors.

	Standardized β	Unstandardized β (CI)		P-value
IFN-γ R^2^ = 0.17				
Sex (m/f)	0.340	459.357 (153.034 to 765.680)		0.004**
BMI (SD)	− 0.318	− 133.285 (− 240.260 to − 26.310)		0.015*
LDL-cholesterol (mmol/L)	0.260	258.203 (71.391 to 445.015)		0.007**
FABP4 (ln(ng/mL))	0.197	190.100 (6.387 to 373.813)		0.043*
Leptin (ln(ng/mL))	0.327	144.755 (23.026 to 266.484)		0.020*
IL-4 R^2^ = 0.11				
QUICKI	0.188	2002.211 (− 61.386 to 3.421)		0.041*
HDL-cholesterol (mmol/L)	0.208	270.409 (84.224 to 3920.198)		0.023*

Multiple linear regression analysis of plasma-induced iNKT cell IFN-γ and IL-4 (n = 114) production in co-culture. Variables entered for backwards selection: Sex, BMI (SD), WHR, fasting glucose, QUICKI, LDL-cholesterol, HDL-cholesterol, triglycerides, hs-CRP, lymphocyte count, monocyte count, FABP4, adiponectin, leptin, chemerin, MCP-1, Cathepsin S. WHR, fasting glucose, triglycerides, hs-CRP, monocyte count, FABP4, leptin, and MCP-1 were first log-transformed. Only significant predictors were reported. *p < 0.05, **p < 0.01.

## Discussion

We investigated whether immunometabolic factors in chronic disease were associated with iNKT cell phenotype and function in a diverse cohort of adolescents with chronic disorders from the *“Cardiovascular Disease in Adolescents with Chronic Disease”* (CDACD) study. The study included adolescents with cystic fibrosis (CF) and characteristic metabolic abnormalities, corrected coarctation of the aorta (CoA) frequently coinciding with hypertension, juvenile idiopathic arthritis (JIA) and a history of chronic inflammation, obesity (OB) and metabolic- and low-grade inflammatory changes, next to healthy adolescents with a history of atrial septal defect (ASD) as a control group. These chronic disorders were selected for their variety of immunometabolic derangements. The role of iNKT cells in disease pathology was only investigated in few of these disorders before. iNKT cells are known to play an important role in the development of obesity and adipose tissue inflammation, for their inflammatory cytokine production in response to adipocyte hypertrophy^7,9^. In cystic fibrosis, an increase in iNKT cells was observed in the lungs of CF mice, yet whether they aggravate or dampen inflammation was not entirely clear^13^. iNKT cells seemed to inhibit inflammation, yet a dual knock-out of the CF transmembrane conductance regulator (CFTR) protein and iNKT cells inhibited inflammation even more, possibly explained by the role of iNKT cells in macrophage and neutrophil recruitment^13^. For ASD, CoA, and JIA patients the role of iNKT cells in disease development has not been studied to date, as far as we are aware.

iNKT cells from obese adolescents showed enhanced expression of the activation marker CD25, and IFN-γ/IL-4 skewing of the cytokine response following *ex-vivo* activation, the latter of which remained significant after correction for multiple testing. Differences in the iNKT cell cytokine response are commonly explained by intrinsic iNKT cell differences, phenotypical heterogeneity, or by differences in antigen presentation or co-stimulation by APCs. In contrast to our expectations, we did not find transcriptional differences underlying the divergent cytokine response. Neither did we observe differences in expression of phenotypic surface antigens such as CD4/CD8, which could otherwise reflect iNKT cell functional heterogeneity^6^. Next, we studied whether differences in circulating APCs could account for the divergent iNKT cell cytokine response. We observed lower CD1d expression on nonclassical monocytes, and higher CD62L expression on B cells in obese adolescents versus controls, which were not significant after correction for multiple testing, and could not explain the observed IFN-γ/IL-4 bias in obese adolescents either. Taken together, the distinct iNKT cell phenotype of obese adolescents was not explained by intrinsic differences, phenotypical diversity, or differences in antigen presentation. Finally, the effect of obesity-associated plasma factors on Th1 skewing of the iNKT cells was studied in a standardized co-culture model. High plasma LDL-cholesterol, fatty-acid binding protein 4 (FABP4), and leptin levels were associated with obesity^14^, and coincided with a higher IFN-γ response of the iNKT cells in co-culture. High HDL-cholesterol and insulin sensitivity (QUICKI) are associated with a healthy weight, and coincided with a higher IL-4 response of iNKT cells. In order to confirm the effect of the lipoproteins on iNKT cell cytokine production, we performed lipoprotein supplementation studies in vitro. We hypothesized that lipoproteins could impact lipid antigen presentation by APC, and pre-treated THP-1 macrophages with LDL or HDL-cholesterol before commencing the co-culture with iNKT cells. Pre-treatment with LDL-cholesterol enhanced the α-GalCer-induced IFN-γ production by the iNKT cells. Moreover, pre-treatment with HDL-cholesterol and LDL-cholesterol enhanced the IL-4 production by the iNKT cells.

The relation between obesity-associated factors and the iNKT cell cytokine response may not come as a surprise. iNKT cells are enriched in human adipose tissue (AT)^15^. Similar to our findings in peripheral blood, AT-resident iNKT cells produce IL-4 under lean conditions, but IFN-γ during obesity, which contributes to adipose tissue inflammation and the development of insulin resistance^7–9^. Recent literature suggests that the lipid-content of the microenvironment is an important determinant of the iNKT cell IFN-γ versus IL-4 cytokine response, even more so than inflammatory stimuli such as TNF-α, IFN-γ, and TLR-2 or TLR-4 ligands^16^. The involved mechanisms remain to be determined, yet may be part of a cascade of metabolic and inflammatory cellular changes induced by lipid loading^17^. In our studies, we specifically observed a relation between circulating lipoprotein levels and the iNKT cell cytokine response. Our in vitro studies suggest an effect of LDL-cholesterol on lipid antigen presentation, as pre-treatment of APC with LDL-cholesterol enhanced the IFN-γ response to α-GalCer. We have not performed further studies into potential mechanisms involved. Our results align with previous studies indicating that specific lipoproteins and their associated apolipoproteins are involved in lipid antigen uptake and processing by APC^18,19^. Alternatively, lipoproteins could affect CD1d stabilization and clustering of CD1d on the APC membrane. Like MHCII, CD1d is localized in lipid raft microdomains in the cell membrane^3,20^. Accumulation of cellular cholesterol due to lipoprotein uptake and processing by APC may dysregulate lipid raft turnover, leading to prolonged immune cell signaling^21^. Since only the iNKT cell IFN-γ response, but not the IL-4 response, is dependent on CD1d clustering within lipid rafts, dysregulation of lipid raft turnover may contribute to the enhanced α-GalCer-induced IFN-γ response that we observed upon LDL-cholesterol loading of the APC^3^. On the other hand, LDL and HDL also induced the IL-4 response of iNKT cells, with and without α-GalCer. The lipoprotein effects on the iNKT cell IL-4 response are in accordance with previous studies. Defective uptake of lipoproteins coincided with a diminished iNKT cell IL-4 response in previous mouse studies^22^, suggesting that lipoproteins can enhance iNKT cell IL-4 production. Moreover, co-culture studies of macrophages and CD4^+^ T cells showed that supplementation of HDL-cholesterol induced the IL-4 production of CD4^+^ T cells^23^. Potential mechanisms involved still require further elucidation. Based on our in vitro studies, lipoproteins appear to affect iNKT cell cytokine production via modulation of the APC, for the lipoproteins were washed away before commencing the coculture with the iNKT cells. Our studies however do not preclude additional direct effects of lipoproteins on the iNKT cells. The uptake of lipoproteins by T cells can affect their intracellular sterol metabolism, which is closely linked to T-cell proliferation and activation^24,25^. Lipoproteins may impact iNKT cell function in a similar fashion, though that has not been studied so far. Further studies are needed to detail the distinct effects of lipoproteins on APC and iNKT cells.

Next to lipoproteins, FABP4 and leptin were identified as predictors of the iNKT cell IFN-γ response. Both adipokines are upregulated during obesity and are associated with sequelae such as insulin resistance, type II diabetes, and cardiovascular disease^26,27^. FABP4 has been implicated in adipose tissue inflammation and inflammatory macrophage polarization^26^, and may contribute to iNKT cell polarization in that manner. Leptin can polarize conventional T cells towards a pro-inflammatory cytokine response, by direct activation of the leptin receptor and subsequent JAK-STAT signaling^28^. iNKT cells also express the leptin receptor, but recent studies indicate that leptin induces iNKT cell anergy rather than polarization^29,30^. The iNKT cell response to leptin is known to depend on leptin receptor expression, which was not evaluated in the CDACD cohort. Follow-up studies are needed to assess iNKT cell leptin receptor expression, and to study the relation between circulating leptin levels, leptin receptor expression, and iNKT cell cytokine production in more in detail.

Upon exclusion of the obese adolescents, leptin and LDL-cholesterol levels were still associated with IFN-γ/IL-4 cytokine skewing. At the same time, multivariable linear regression analysis showed a negative association of BMI standard deviation (SD) with iNKT cell IFN-γ production. These two observations may reflect the same phenomenon. A higher BMI does not always coincide with obesity-associated inflammation and dyslipidemia, especially in children and adolescents^31^. The accumulation of subcutaneous adipose tissue, for example, contributes to a higher BMI but is not associated with inflammation and dyslipidemia^32^. Vice versa, obesity-associated plasma factors such as FAPB4, leptin, and LDL-cholesterol represent metabolically affected adolescents with chronic disease, who are often not obese at all. Further research is needed to unravel the effects of distinct obesity-associated plasma factors and adipose tissue homeostasis on circulating iNKT cell phenotype and function.

Limitations of our study include the use of peripheral blood iNKT cells, while tissue-resident iNKT cells might be equally or even more relevant for the development of immunometabolic disease. Second, there were sex differences between patient groups. These are partly explained by disease epidemiology, since CoA is more common in males, and ASD is more common in females^33^. To ensure that sex differences did not affect our main outcomes, sex was included in the multivariable regression analysis (Table 2). The IFN-γ/IL-4 ratio (Fig. 1E) was not different between the sexes. Third, although clinical symptoms of acute inflammation were an exclusion criterium for the study, six participants showed slightly elevated CRP levels (10–21 mg/L), which corresponded with hs-CRP levels of 28.29–42.69 mg/L. In order to account for potential effects of acute inflammation on iNKT cell function, we repeated our main analyses without these six participants, which did not impact our main outcomes (data not shown). It is therefore unlikely that acute inflammatory effects account for our observations. A final limitation of our study is that we only performed RNA sequencing of iNKT cells in a subset of patients. Analysis of the full set of obese versus healthy subjects would have increased statistical power to detect differences between groups. Moreover, RNA sequencing was performed in unstimulated iNKT cells, to study the effect of their recent exposure to circulating immunometabolic factors in vivo. The addition of RNA sequencing upon ex vivo antigenic stimulation of the iNKT cells, and possibly even single-cell RNA sequencing, may have provided more insights in subtle transcriptional differences, also upon activation.

## Conclusions

Obese adolescents showed enhanced expression of the activation marker CD25 on circulating iNKT cells, and IFN-γ/IL-4 cytokine skewing following ex-vivo activation of the circulating iNKT cells. The Th1 skewing of iNKT cells was associated with immunometabolic factors including LDL-cholesterol, leptin, and FABP4, both in obese and non-obese adolescents with chronic disease. Conversely, favorable immunometabolic factors such as HDL-cholesterol and insulin sensitivity (QUICKI) were associated with higher IL-4 production of the circulating iNKT cells. In vitro supplementation studies corroborated the effects of lipoproteins on iNKT cell cytokine production. Our findings suggest that circulating immunometabolic factors such as lipoproteins may be involved in the Th1 cytokine skewing of iNKT cells in immunometabolic disease.

## Methods

### Study design and population

The CDACD study cohort included 114 adolescents aged 12–18. The cross-sectional and observational study was performed at the Wilhelmina Children’s Hospital in Utrecht, the Netherlands, between April 2017 and June 2019. The study population included patients with cystic fibrosis (CF, n = 24), corrected coarctation of the aorta (CoA, n = 25), rheumatoid factor negative polyarticular or extended oligoarticular juvenile idiopathic arthritis (JIA, n = 20), obesity (OB, n = 20), and healthy adolescents with a corrected atrial septal defect as control group (ASD, n = 25). Obesity was defined as a body mass index > 30 kg/m2 projected to the age of 18 years, according to the international Obesity Task Force^34^. Exclusion criteria for all participants were acute illness, mental retardation, pregnancy, or contraindications for MRI with gadolinium contrast. Written informed consent was obtained from all participants, and their parents/guardians when appropriate. The study complies with the Declaration of Helsinki and ethical approval was obtained from the institutional Medical Research Ethics Committee of the University Medical Center Utrecht (protocol number 16–589).

### Clinical characteristics and immunometabolic profiles

BMI (SD) and waist-to-hip ratio were measured following established protocols^14^. Fasting glucose, fasting insulin, fasting lipid profile, and blood count were measured by the diagnostic laboratory of the University Medical Center Utrecht following local clinical protocols. Adipokines (FABP4, adiponectin, leptin, chemerin, MCP-1, cathepsin S) and hs-CRP were measured using multiplex immunoassay (Luminex) by the Luminex core facility of the University Medical Center Utrecht. Luminex-based hs-CRP measurements are 2–3 times higher than routine diagnostic CRP measurements due to assay characteristics^35^.

### Flow cytometry

Peripheral blood mononuclear cells (PBMC) were isolated using Ficoll density gradient centrifugation and stored in liquid nitrogen. iNKT cells were stained with Fixable Viability dye eFluor 507 (eBioscience) in PBS and subsequently stained with surface antibodies CD3, CD4, CD8, CD25, and a CD1d tetramer (NIH) in FACS buffer (PBS with 2% FCS [Invitrogen] and 0,1% sodium azide [Sigma-Aldrich]). The iNKT cell gating strategy is shown in supplementary figure S5 and the specific antibodies used are listed in supplementary table S2. When relevant, iNKT cells were fixed and permeabilized using Fixation and Permeabilization buffer (eBioscience), and stained with IFN-γ, IL-4, IL-17A, and Ki-67. Antigen presenting cells were stained separately. After exclusion of doublets, B cells were gated as CD19^+^ lymphocytes. Dendritic cells were gated after exclusion of T cells, B cells, monocytes and NK cells as CD3^−^, CD19^−^, CD14^−^, CD16^−^, CD56^−^, as HLA-DR^+^CD11c^+^, following the gating strategy in previous publications^36^. Monocytes were gated after exclusion of T cells and B cells, as HLA-DR^+^ CD14^+^ positive cells, followed by subgating of classical monocytes (CD14^++^CD16^-^), intermediate monocytes (CD14^++^CD16^+^), and nonclassical monocytes (CD14^+^CD16^++^), following previous publications^14,37^. For intracellular-cytokine staining, cells were stimulated for four hours using PMA (25 ng/mL, MP Biomedicals), ionomycin (1 ug/mL, Calbiochem), and GolgiStop (1/1500, BD). Data were analyzed using Flow Jo (BD). Samples with less than 20 iNKT cells were excluded from analyses to allow subsequent subgating, resulting in analysis of 17 samples in the ASD group, 17 in the CF group, 22 in the CoA group, 18 in the JIA group, and 17 in the obese group.

### Ex-vivo activation assay

1,5–2*10^6 PBMC were cultured in a 24-well plate in 1 mL RPMI culture medium (Gibco) supplemented with 10% FCS, 100 U/mL penicillin- streptomycin, 2 mM L- glutamine, and 100 U/mL IL-2. Cells were stimulated once with 1 ug/mL alpha-Galactosylceramide (Avanti Lipids). Supernatants of 109 samples (ASD, n = 25; CF, n = 24; CoA, n = 24; JIA, n = 18; and obese, n = 18) were obtained at day 11 and stored at − 80 °C until measurement of IFN-γ, IL-4 and IL-17 using multiplex immunoassay (Luminex).

### Low-input RNA sequencing

iNKT cells from seven obese and seven age- and sex matched ASD controls were sorted directly into Trizol (Invitrogen), snap-frozen and stored at − 80 °C. RNA isolation was performed following the Trizol manufacturer’s protocol and low input libraries were prepared using the CEL-Seq2 sample preparation protocol^38^. Sequencing reads were mapped against the reference human genome (hg19, NCBI37) using BWA^39^, and normalized per million reads. As quality control, only matched samples with expression of > 10.000 genes were used and low expressed genes with less than 1 count or 1 read per million (RPM) across samples were removed. Differentially expressed genes were identified using the DESeq2 package from Bioconductor in R (www.r-project.org). Pathways analysis was performed using ToppGene^40^. Gene set enrichment analysis (GSEA) was performed using datasets published in the Molecular Signatures Database (MSigDB). Heatmaps were prepared using RPM Z-scores with heatmap.2 in R.

### Co-culture experiments

THP-1 cells with stable overexpression of CD1d were a kind gift of M. Salio^41^, and were plated at 50.000 cells/well in 100 uL RPMI culture medium (Gibco) supplemented with 100 U/mL penicillin- streptomycin and 2 mM L- glutamine, but without FCS, in a flat bottom 96-well plate. Culture medium was supplemented with 25% human plasma from patients from the CDACD study, which was obtained from patients in sodium-heparin tubes after spinning for 10 min at 1200 RPM and stored at − 80 °C. After 24 h, 80.000 iNKT cells from a healthy donor-derived short-term iNKT cell line were added to each well and co-cultured for another 24 h, after which supernatants were collected of all 114 samples for IFN-γ and IL-4 measurements using ELISA (Biolegend). The short term iNKT cell line was derived from a healthy donor following established protocols^42^. For comparison, THP-1 cells only (n = 6) and iNKT cells only (n = 4), stimulated with a random selection of patient plasma samples were included.

### Lipoprotein supplementation studies

Lipoproteins were isolated from human serum (NHS blood donation service, London) using established ultracentrifugation protocols^43^. THP-1 macrophages were generated by differentiation of 100.000 THP-1 monocytes using 100 ng/ml PMA (Sigma) for 48 h. Upon differentiation, THP-1 macrophages were treated with 30 μg/ml human LDL or HDL with or without 100 ng/ml α-GalCer or α-GalCer alone overnight. THP-1 cells were washed with phosphate-buffered saline before adding 50.000 human iNKT cells for a 24-h co- culture. Supernatants were harvested and analyzed for human IFN-γ and IL-4 levels using ELISA (Biolegend).

### Statistics

Normally distributed variables are presented as mean and standard deviation and groups were compared using ANOVA, followed by post-hoc testing against controls (ASD group) using independent *t* tests. In case of nonnormality, median and interquartile range are reported and groups were compared using Kruskal–Wallis, followed by post-hoc testing against ASD controls using Mann–Whitney U tests. Bonferroni correction for multiple testing was applied when appropriate. For backward multivariable linear regression analysis (n = 114) predictor variables were natural-logarithmically transformed if their skewness was > 1. Statistical analyses were performed using IBM SPSS Statistics 24 and Prism 8 for MacOS (Graphpad Software).

## Supplementary Information

Supplementary Information.

## Data Availability

Data are available from the corresponding author upon request.
