# Supplementary material for: Immunometabolic factors in adolescent chronic disease are associated with Th1 skewing of invariant Natural Killer T cells
Source: Sci Rep. 2021 Oct 11;11:20082. doi: 10.1038/s41598-021-99580-7 (PMC8505552; doi:10.1038/s41598-021-99580-7)
Supplement: Supplementary file 1 — Supplementary Information. [file 41598_2021_99580_MOESM1_ESM.docx]

***Supplemental appendix***

**Immunometabolic factors in adolescent chronic disease are associated**

**with Th1 skewing of invariant Natural Killer T cells**

**Contents**

1. Supplemental figures and tables Page 2-4
2. Supplemental methods Page 5

**Supplemental figures and tables**

**Supplemental figure S1**: iNKT cell proliferation following aGalCer activation. ****p<0.0001.

A

B

C

**Supplemental figure S2**: A) CD1d expression on antigen presenting cells (APC) and B) CD62L and C) CD11b expression on APC. Disease groups were compared using Kruskal-Wallis, followed by post-hoc Mann-Whitney U comparisons against ASD controls, *p<0.05. Differences were not significant after Bonferroni correction for multiple testing.

**Supplemental figure S3:** Heatmap displaying the top differentially expressed genes (selected based on nominal p-value <0.01).

**Supplemental table S1:** Multivariable linear regression analysis excluding the obese population

| IFN-γ R^2^ = 0.23 | | | |
| --- | --- | --- | --- |
|  | **Standardized β** | **Unstandardized β (CI)** | **P-value** |
| Sex (m/f) | 0.247 | 336.717 (10.036- 663.397) | 0.044* |
| BMI (SD) | -0.381 | -239.562 (-376.817- -102.308) | 0.001** |
| LDL-cholesterol (mmol/L) | 0.341 | 343.445 (134.321- 552.570) | 0.002** |
| Triglycerides (ln(mmol/L)) | -0.215 | -351.469 (-678.006- -24.932) | 0.035* |
| Leptin (ln(ng/mL)) | 0.261 | 132.059 (7.101- 257.018) | 0.039* |

| IL-4 R^2^ = 0.11 | | | |
| --- | --- | --- | --- |
|  | **Standardized β** | **Unstandardized β (CI)** | **P-value** |
| QUICKI | 0.205 | 2568.719 (24.684- 5112.754) | 0.048* |
| HDL-cholesterol (mmol/L) | 0.245 | 321.679 (59.067- 584.292) | 0.017* |

Variables entered for backwards selection: Sex, BMI (SD), WHR, fasting glucose, QUICKI, LDL-cholesterol, HDL-cholesterol, triglycerides, hs-CRP, lymphocyte count, monocyte count, FABP4, adiponectin, leptin, chemerin, MCP-1, Cathepsin S. WHR, fasting glucose, triglycerides, hs-CRP, monocyte count, FABP4, leptin, and MCP-1 were first log-transformed. Only significant predictors were reported. *p<0.05, **p<0.01.

A

B

**Supplemental figure S4:** Plasma-induced iNKT cell cytokine production in co-culture measured using ELISA, color-coded per group: ASD: blue dots; CF: orange triangles; CoA: green diamonds; JIA: black- lined yellow circles; OB: red squares. A) IFN-γ production corresponded with LDL-cholesterol levels in plasma (n=114, Pearson’s R=0.222, p=0.017) but not HDL-cholesterol (n=114, Pearson’s R=0.100, p=0.288). B) IL-4 cytokine production corresponded with HDL-cholesterol levels in plasma (n=114, Pearson’s R=0.206, p=0.029) but not LDL-cholesterol (n=114, Pearson’s R=0.115, p=0.224).

**Supplemental methods**


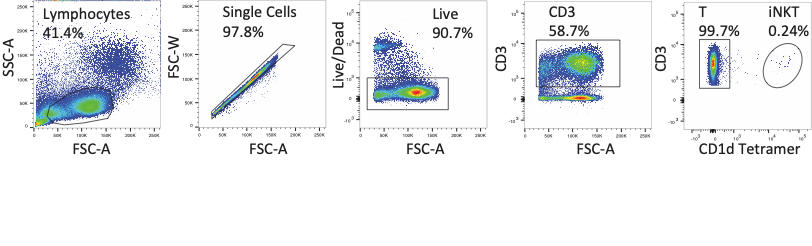


**Supplemental figure S5:** Representative gating strategy for iNKT cells.

**Supplemental table S2:** List of antibodies used for the iNKT staining (left) and APC staining (right).

| CD19-BV711 (Biolegend) |
| --- |
| CD3-APC (BD) |
| CD19-BV711 (Biolegend) |
| CD14-APC-Cy7 (BD) |
| CD16-V500 (BD) |
| CD56-PerCP-Cy5.5 (BD) |
| HLA-DR-FITC (eBioscience) |
| CD11c-V450 (BD) |
| CD11b-AF700 (BD) |
| CD1d-PE (BD) |
| CD62L-PE-Cy7 (Biolegend) |

| Fixable Viability dye eFluor 507 (eBioscience) |
| --- |
| CD3-AF700 (Sony Biotechnology) |
| CD1d-tetramer-BV421 loaded with PBS-57 (NIH tetramer core facility) |
| CD4-PE-Cy5 (BD) |
| CD8-APC-Cy7 (BD) |
| CD25-PE-Cy7 (BD) |
| IFN-γ-PerCP-Cy5.5 (eBioscience) |
| IL-4-BV711 (BD) |
| IL-17A-FITC (eBioscience) |
| Ki-67-AF647 (BD) |
